# Short-Term Outcomes and Cosmetic Results of Pre-pectoral Implant-Based Breast Reconstruction Using Braxon® Mesh: A Systematic Review of Post-operative Complications

**DOI:** 10.7759/cureus.77810

**Published:** 2025-01-22

**Authors:** Isha Karwasra, Abhijeet Beniwal, Matei Dordea

**Affiliations:** 1 Surgery, University Hospital North Tees and Hartlepool NHS Foundation Trust, Stockton-on-Tees, GBR

**Keywords:** breast reconstruction surgery, cosmetic surgeries, implant-based breast reconstruction, oncoplastic breast surgery, pre-pectoral reconstruction

## Abstract

The technique of implant immediate breast reconstruction has been revived since the advent of acellular dermal matrices (ADM). The traditional technique involving sub-pectoral implant placement is being replaced by the re-emerging technique of muscle-sparing pre-pectoral implant placement due to the availability of ADM, which can wrapped around the implant, thereby obviating the need for any additional tissue cover. Braxon® (DECO Med s.r.l., Marcon, Italy), a novel ADM, specifically designed for breast reconstruction, is gaining popularity among surgeons in the UK and Europe. Its early outcomes seem promising; however, the literature to back its utility is still scarce. Hence, there is a need to gather and compile the existing evidence to inform the current clinical practice.

A systematic review was carried out for all the original studies reporting the outcomes of pre-pectoral implant breast reconstruction with Braxon® through MEDLINE and CINAHL databases. Studies were selected and analyzed based on their level of evidence, inclusion and exclusion criteria for pre-pectoral implant breast reconstruction, and outcomes in terms of complication rates and cosmesis.

Six studies (742 breast reconstructions in 600 patients) were identified for the review. All of the studies were level IV evidence case series, reporting outcomes for at least three months post-operative follow-up. All except one study mentioned patient selection criteria based on Association of Breast Surgery (ABS) and British Association of Plastic Reconstructive and Aesthetic Surgeons (BAPRAS) guidelines. Pooled complication rates showed that total 209 (28.2%) breast reconstructions had some form of complication; major complication rate of 98 (13.2%), return to theater 90 (12.1%), implant loss 48 (6.5%), minor complications 111 (15%), seroma 74 (10%), hematoma 29 (3.9%), Red breast syndrome 38 (5.1%), infection 26 (3.5%), necrosis 26 (3.5%), and wound dehiscence 24(3.2%).

The short-term pooled complication rates of pre-pectoral implant breast reconstruction with Braxon® are low and comparable with those of the sub-pectoral technique. Although preliminary data are promising; however, the long-term outcomes are yet to be analyzed in future studies.

## Introduction and background

Breast cancer is not only the most common female cancer but is also the most common cancer overall worldwide, closely followed by lung cancer. As per the estimates from GLOBOCAN 2020, breast cancer is the overall leading cancer in terms of incidence, with 2.3 million cases of new breast cancer diagnoses in the year 2020 [1]. The rising incidence of breast cancer is offset by the rapidly evolving treatment strategies. The advancements in breast cancer management have not only evolved around improving survival and cure but have also come to address issues impacting the quality of life after the treatment in breast cancer survivors.

Nowadays, it is standard practice to offer immediate breast reconstruction (IBR) to every patient undergoing mastectomy. The traditional technique of implant reconstruction involves placement of implant in a muscular pocket created by mobilizing the pectoralis major muscle [2]. However, there were some demerits of this technique, including pain and discomfort from muscle detachment and significant animation deformity, especially in young women. This led to the subsequent development of pre-pectoral implant-based breast reconstruction [3]. Skin-sparing and nipple-sparing mastectomy with implant-based immediate breast reconstruction has been deemed as oncologically safe, which has allowed surgeons to give cosmetically optimal results [4].

Recently, there has been an ongoing transition from sub-pectoral implant placement to a pre-pectoral implant placement due to certain perceived advantages, mainly to avoid morbidity associated with detachment of the pectoralis muscle. This has been the consequence of the use of acellular dermal matrix (ADM) in breast reconstructions. Acellular dermal matrices (ADMs) are biotechnologically engineered matrices derived from human, bovine, or porcine tissues, which have been processed to remove the cellular antigens capable of eliciting an immune response in the host. They only retain the structural matrix that acts as a scaffolding to allow angiogenesis and tissue regeneration. The various commercially available ADMs include Strattice® (Allergan Aesthetics, Irvine, California), Surgimend® (Integra LifeSciences Corporation, Princeton, New Jersey), Native® (LifeCell, Irvine, California), Veritas® (Deerfield, Illinois), and Braxon® (DECO Med s.r.l., Marcon, Italy). ADM allows pre-pectoral implant placement directly under the skin flap by providing coverage over the implant without the need to detach the pectoralis muscle [5]. Consequently, the post-operative pain from muscle detachment is avoided, leading to quicker post-operative recovery [6].

A systematic review was conducted by Salibian et al. in 2016 to analyze the outcomes of subcutaneous (pre-pectoral) implant-based breast reconstruction using ADM and meshes [7]. They included six studies, representing a total of 186 breast reconstructions identified for review, most of which were case series, contributing to level 4 evidence. ADM was used in 60.2% of reconstructions and mesh in the rest. Their pooled complication rates were low: seroma 2.9%, hematoma 2.3%, major infection 1.2%, complete nipple-areola complex (NAC) necrosis 1.1%, partial nipple-areola complex necrosis 4.5%, skin flap necrosis 1.8%, wound healing complications 2.3%, implant loss 4.1%, and capsular contracture rate 1.2%. They concluded that pre-pectoral implant breast reconstruction with ADM was a promising technique and was not associated with high complication rates [7].

The use of Braxon® has been fast spreading among breast surgeons in the UK since its introduction. Derived from the porcine dermis, it comes as a 0.6 mm-thick pre-shaped mesh, which is stitched together to completely wrap the implant ex-vivo (Braxon® ADM, Raise Healthcare, Sutton Coldfield, UK). Although the literature is scarce, the early evidence suggests promising outcomes with low complications and satisfactory cosmetic results in terms of shape, ptosis, and softness.

Pre-pectoral implant-based breast reconstruction with placement of breast implant in the subcutaneous plane was developed more than four decades ago. However, interestingly, the traditional practice of implant-based breast reconstruction happens to be the sub-pectoral technique of implant placement, which evolved as a method to overcome complications seen with the former [8]. This was because the subcutaneous implant placement technique was associated with complications like capsular contracture, implant mal-positioning, and implant loss secondary to infections and other wound complications.

While there is ample published literature on the use of ADMs, in general, in pre-pectoral implant reconstructions, the reported outcomes of Braxon® ADM are limited, owing to its relatively recent development [9]. Hence, there is a need to gather and compile the existing evidence to inform the current clinical practice.

We performed a systematic review to establish the short-term safety outcomes in terms of complication rates (implant loss, infection, reoperation, and re-admission at three months) of pre-pectoral implant-based breast reconstruction with Braxon® mesh.

## Review

Methodology

A comprehensive systematic review was conducted to search and synthesize evidence on the post-operative outcomes of pre-pectoral implant reconstructions with Braxon® mesh. MEDLINE and CINAHL databases were searched in May 2022 for all the UpToDate original studies reporting the outcomes of pre-pectoral implant-based breast reconstruction with Braxon®. This study was conducted in accordance with the Preferred Reporting Items for Systematic Reviews and Meta-Analyses (PRISMA) guidelines to ensure transparency and accuracy in reporting. The PRISMA checklist was followed throughout the review process, including study selection, data extraction, and synthesis of findings. Studies were selected and analyzed based on their level of evidence, inclusion and exclusion criteria for pre-pectoral implant-based breast reconstruction, and outcomes in terms of complication rates and cosmesis. The search was conducted using the following keywords: “Pre-pectoral”, “Implant reconstruction”, “Muscle sparing”, “Sub-cutaneous breast reconstruction”, “Braxon®”, and “ADM mesh”.

Headings were screened to include all studies reporting outcomes of pre-pectoral implant breast reconstruction with Braxon®. Abstracts of the selected studies were reviewed to include those studies that reported the primary post-operative outcomes (primary outcomes including complication rates and secondary outcomes including cosmesis) of pre-pectoral breast reconstruction with Braxon® for a minimum 90-day period. Those reporting outcomes of other types of reconstructions, or those not reporting the primary and secondary outcomes of reconstruction with Braxon®, any case reports, abstracts, non-English articles, or review articles were excluded. In addition, literature cited by the manufacturer, Raise Healthcare, was searched, along with references cited in the selected studies on Braxon®.

The quality of the included studies was further analyzed by going through the full text. Each full-text article was critically appraised. Data were extracted on study type, patient demographics, and post-operative complication rates. Complications were analyzed as major complications (necessitating hospital admission, return to theater, or need for IV antibiotics), implant loss, minor complications, seroma, necrosis of skin flaps and NAC, infection, wound dehiscence, and Red breast syndrome. Reports of capsular contracture and cosmetic outcomes were also analyzed. The extracted data were combined to derive pooled complication rates.

Results

**Figure 1:**
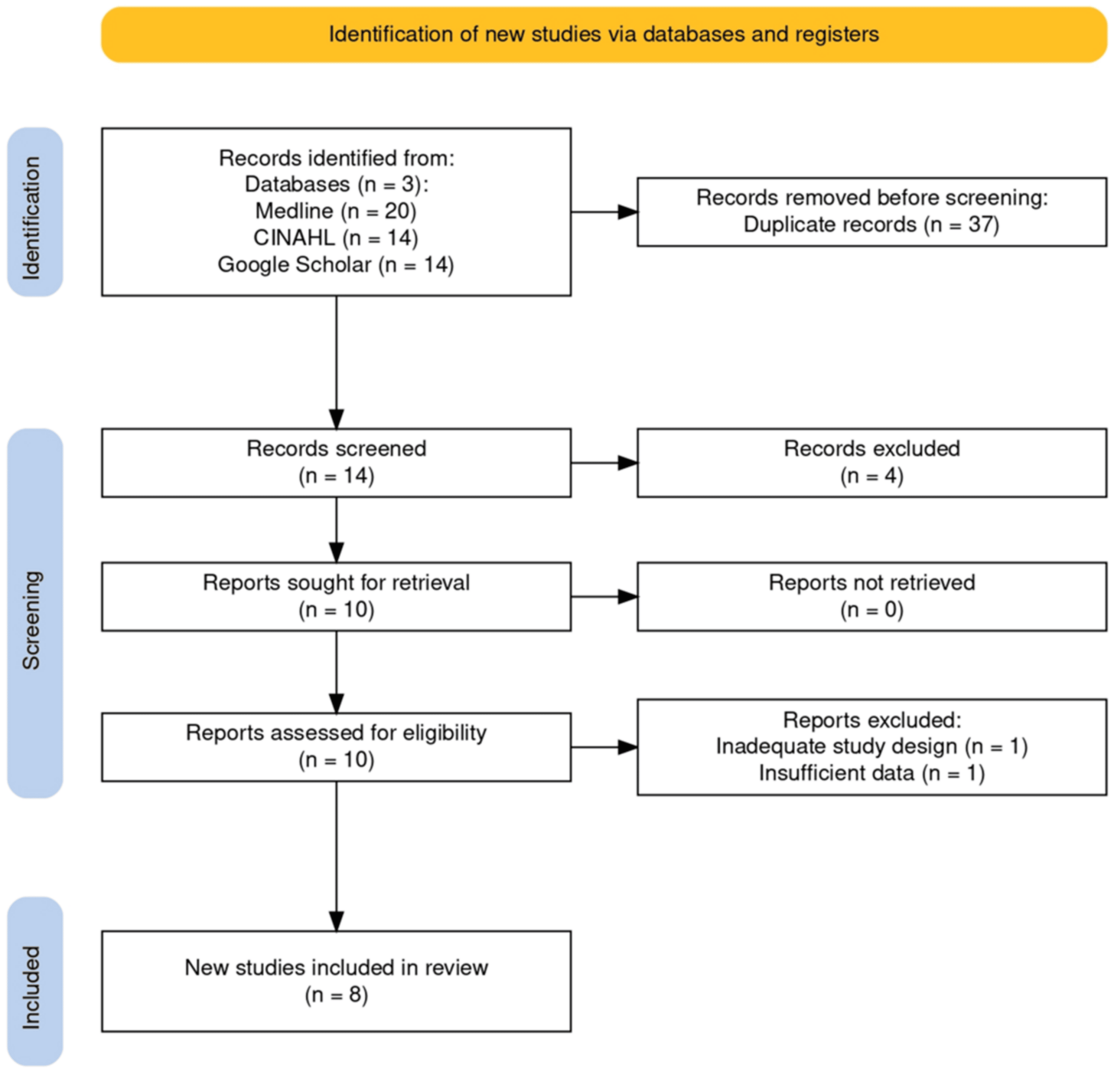
PRISMA flow diagram. PRISMA: Preferred Reporting Items for Systematic Reviews and Meta-Analyses.

A total of 14 studies were screened following the search. Finally, eight studies were identified as deemed appropriate for this review, and all of these studies were level IV evidence case series. No randomized control trials were identified for Braxon®. There were no studies found comparing the outcomes of pre-pectoral implant-based breast reconstructions using Braxon® and those of sub-pectoral implant-based breast reconstruction (Figure 1).

All eight studies were based in European centers, primarily in the United Kingdom and Italy, and a few in Spain and Poland. These studies were published over a span of five years, from 2017 through 2021, reflecting its recent introduction and growing interest among surgeons in the UK and Europe. Table 1 summarizes the studies included in this systematic review.

**Table 1:** Summary of included studies.

Study	Year	Study design	Patients	Number of reconstructed breasts	Unilateral	Bilateral	Median follow-up (in months)
Jafferbhoy et al. [5]	2017	Case series (level IV)	64	78	50	14	9.98
Berna et al. [6]	2017	Case series (level IV)	19	25	13	6	14±4.60 (7-20)
Masia et al. [10]	2020	Case series (level IV)	1186	1450	922	264	22.7 (3-75.7)
Chandarana et al. [11]	2019	Case series (level IV)	324	406	242	82	9.7 (3-35)
Bojanic et al. [12]	2021	Case series (level IV)	16	22	10	6	19
Mura et al. [13]	2021	Case series (level IV)	98	111	85	13	9
Vidya et al. [14]	2017	Case series (level IV)	51	60	42	9	16.4 (8-25)
Vidya et al. [15]	2017	Case series (level IV)	79	100	58	21	17.9±3.6 (9.5-25.7)

All of the included studies had a minimum follow-up of at least three months (90 days) and reported short-term complication rates, including seroma, infection, hematoma, wound complications, Red breast syndrome, and implant loss rates. Most studies mentioned satisfactory cosmetic outcomes in their respective follow-ups; however, there was no objective assessment of the same through a patient-reported outcome measures (PROMS) tool such as Breast Q.

The largest case series so far has been reported through the implant-based breast reconstruction evaluation (iBRA) study, which was an international multicenter audit involving a total of 1450 breast reconstructions in 1186 patients across 30 centers in the EU and UK [10]. The next largest case series was represented by Chandarana et al., with 324 patients and a total of 406 breast reconstructions [11]. The smallest case series was that of Bojanic et al., representing 22 reconstructions in 16 patients [12]. Patient selection for most studies was based on similar inclusion criteria, as per the British Association of Plastic Reconstructive and Aesthetic Surgeons (BAPRAS) guidelines. These included a BMI less than 30 kg/m², no previous radiotherapy, an estimated mastectomy weight of less than 600 g, and a good subcutaneous layer (assessed by a pinch test >1 cm on the upper and medial quadrants). In addition to these, Mura et al. mentioned a few more criteria, such as small to medium-sized breasts with mild to moderate ptosis and the absence of relative contraindications like smoking, uncontrolled diabetes, immune deficiency, morbid obesity, and locally advanced breast cancer [13]. There was no explicit mention of inclusion criteria by Bojanic et al. [12]. While Mura et al. have not described the patient demographics of their series, the mean age and mean BMI are similar in the rest of the studies [13]. Patient demographic details of each study are given in Table 2.

**Table 2:** Study-wise patient demographics. RT: radiotherapy.

Study name	Mean age (years)	Mean BMI (kg/m^2^)	Mean implant volume (cc)	Smokers (number of patients)	Diabetes (number of patients)	Post-op RT (number of patients)
Berna et al. [6]	52±6.73 (48-63)	25.4±2.99 (22.8-28.5)	N/A	2	0	N/A
Vidya et al. [14]	55 (28-71)	26.4 (20.3- 34.8)	360 (175-480)	0	N/A	0
Vidya et al. [15]	55.8±13.6 (28-74)	24.4±4.0 (20.3-27.6)	336.6±78.5 (205-525)	N/A	N/A	3
Jafferbhoy et al. [5]	50	25.7	364.75 (200-535)	13	2	N/A
Chandarana et al. [11]	49 (29-82)	25 (18-43)	370 (105-685)	15	7	62
Masia et al. [10]	52.4±11.4 (17‐87)	23.9±4.1 (15.6‐40)	349.2±112.6 (100‐685)	120	28	153
Mura et al. [13]	N/A	N/A	N/A	N/A	N/A	N/A
Bojanic et al. [12]	43.5 (35-55)	25.9 (21.7-50)	N/A	1	0	N/A

Berna et al. published their results on 19 patients (a total of 25 implant reconstructions), of which six had bilateral and 13 had unilateral pre-pectoral implant-based breast reconstructions [6]. The first ten breast reconstructions in their series were done initially using a 0.9-mm-thick porcine ADM with preservatives. For the next 15 reconstructions, they switched to the 0.6-mm-thick Braxon® ADM. They reported implant loss in 3 (12%) reconstructions with the first type of ADM, which were due to seroma 2 (8%) and infection 1 (4%). However, with the 0.6-mm-thick Braxon® ADM, only minor complications were noted, such as seroma 2 (8%), which was treated by aspiration. Interestingly, there were no major complications attributed to the use of Braxon®, with no resulting implant loss. They further reported good cosmetic outcomes with Braxon® ADM in terms of symmetry, natural-looking shape, ptosis, and softness of the reconstructed breast. There were no reports of post-operative pain, unlike that seen in sub-pectoral implant reconstruction. The same authors also published the long-term outcomes of their first ten pre-pectoral implant-based reconstructions using Braxon® ADM, performed between November 2012 and January 2013. They again reported good cosmetic outcomes, with no cases of capsular contracture in any of the ten patients after four years of follow-up [6].

The first nationwide audit was the National Mastectomy and Breast Reconstruction Audit 2009 [16]. It analyzed the outcomes of over 3000 patients who underwent implant-based breast reconstruction between 1st January 2008 and 31st March 2009. They reported an overall complication rate of 441 (14.7%), a return to theater rate of 138 (4.6%), and explantation rate of 270 (9%), with sub-pectoral implant placement.

The next and the most recent audit is the implant-based breast reconstruction evaluation (iBRA) study, which evaluated the outcomes of 2655 implant-based breast reconstructions in 2108 patients, operated between 1st February 2014 and 30th June 2016, across 81 units in the UK. Of all patients studied, only 42 (2%) had pre-pectoral implant reconstruction. Overall, the re-admission and return to theater were 380 (18%) each, and 189 (9%) patients had implant loss within 90 days [17].

Vidya et al. published the results of a total of 51 muscle-sparing pre-pectoral implant-based breast reconstructions using Braxon® (42 unilateral and nine bilateral), which were performed between April 2014 and September 2015 at two centers in the UK [14]. The median follow-up period of their study was 16.4 months, and they reported complications in six cases, including seroma formation 4 (6.7%), implant loss secondary to wound infection 1 (1.7%), and uncomplicated superficial wound dehiscence, which was re-sutured 1 (1.7%). The same authors also reported the outcomes of the European prospective study on pre-pectoral implant breast reconstruction with Braxon® ADM. This study enrolled 79 patients (a total of 100 breast reconstructions) across Europe between April 2014 and August 2015. Patient selection was done through a uniform protocol as per the joint guideline from the Association of Breast Surgery (ABS) and the British Association of Plastic Reconstructive and Aesthetic Surgeons (BAPRAS) for the use of ADM in breast surgery. Of the 79 patients, 21 had bilateral reconstructions and the rest had unilateral reconstructions. With a mean follow-up of 17.9 months, they reported one case each of nipple necrosis 1 (1%) and wound breakdown 1 (1%), leading to implant loss in these two patients (2%). Other complications included hematoma in 2 (2%), wound dehiscence in 3 (3%), and seroma in 5 (5%) of cases. Overall, good cosmetic outcomes were reported, denoted by softness to touch and natural movement of the reconstructed breasts. No patient was reported to experience post-operative pain or reduced movements of the pectoralis major muscle [15]. As the patients included in this study were from the same time frame as those of the previous publication by the same authors, it is presumed that there must have been an overlap of the included patients.

Jafferbhoy et al. conducted a prospective study from December 2015 to October 2016 on all patients who underwent mastectomy followed by an implant-based immediate breast reconstruction using Braxon® ADM in three different breast units in the UK [5]. They analyzed a total of 78 breast reconstructions done in 64 patients, with a mean age of 50 years and a mean BMI of 25.7 kg/m². Their median follow-up time was 9.98 months. The complications reported by them included seroma in 15 (19.2%) of patients, erythema (Red breast syndrome) treated with antibiotics in 19 (24.4%), and implant loss in 8 (10.2%) of patients. They did not report any case of skin or NAC necrosis, and their overall major complication rate was 14 (17.9%). Their univariate analysis showed that perioperative complications and implant loss had a significant association with bilateral reconstructions.

Chandarana et al. reported the outcomes of a multicenter audit of pre-pectoral breast reconstructions with Braxon® ADM between January 2015 and December 2017 [11]. They included 18 centers across the UK, reporting the outcomes of a total of 406 breast reconstructions done in 324 patients. The post-operative outcomes were recorded for a minimum of 90 days, with a median follow-up of 9.65 months. Almost 62 (15.3%) of patients had a major complication, including implant loss, seen in 20 (4.9%) of patients within the first 90 days. Another 6 (1.5%) patients had delayed loss of the implant. In multivariable analysis, no factor was identified as significantly correlated with implant loss or other post-operative complications. They concluded that the overall outcomes were satisfactory with the Braxon® ADM mesh. However, they could not evaluate the effects of adjuvant radiotherapy in these patients due to the shorter span of the follow-up.

Of all the studies included, Berna et al. [6] and Vidya et al. [15] reported the lowest incidence of complications. In fact, Berna et al. did not report any major complications with Braxon®, considering the fact that all three major complications in their series were with the initially used 0.9 mm ADM with preservatives [6]. Likewise, Vidya et al. [15] reported a major complication rate of only 2 (2%), including an implant loss rate of 2 (2%), a seroma rate of 5 (5%), and not a single case of infection. This stands in sharp contrast against the complication rates reported by Bojanic et al., where the rate of major complications was 6 (27.2%), with infections in 6 (27.2%), seroma in 8 (36.3%) and implant loss in 6 (27.2%) cases [12]. Table 3 illustrates the complication rates of all the studies included in the review.

**Table 3:** Study-wise complication rates. NAC: nipple-areola complex.

Study name	Major complications	Minor complications	Seroma	Hematoma	Necrosis (skin flap or NAC)	Infection	Implant loss	Wound dehiscence	Red breast syndrome
Berna et al. [6]	3 (12%)	2 (8%)	2 (8%)	0 (0%)	0 (0%)	1 (4%)	3 (12%)	0 (0%)	0 (0%)
Vidya et al. [14]	1 (1.7%)	6 (10%)	4 (6.7%)	0 (0%)	1 (1.7%)	1 (1.7%)	1 (1.7%)	1 (1.7%)	0 (0%)
Vidya et al. [15]	2 (2%)	10 (10%)	5 (5%)	2 (2%)	1 (1%)	0 (0%)	2 (2%)	3 (3%)	0 (0%)
Jafferbhoy et al. [5]	14 (17.9%)	17 (21.8%)	15 (19.2%)	4 (5.1%)	0 (0%)	4 (5.1%)	8 (10.2%)	1 (1.3%)	19 (24.4%)
Chandarana et al. [11]	62 (15.3%)	54 (13.3%)	29 (7.1%)	10 (2.5%)	21(5.2%)	13 (3.2%)	26 (6.4%)	8 (2.0%)	16 (3.9%)
Masia et al. [10]	N/A	N/A	111 (7.7%)	31 (2.1 %)	46 (3.2%)	70 (4.8%)	94 (6.5%)	67 (4.6%)	48 (3.3%)
Mura et al. [13]	11 (9.9%)	23 (20.7%)	15 (13.5%)	12 (10.8%)	3 (2.7%)	2 (1.8%)	3 (2.7%)	13 (11.7%)	0 (0%)
Bojanic et al. [12]	6 (27.2%)	5 (22.7%)	8 (36.3%)	1 (4.5%)	1 (4.5%)	6 (27.2%)	6 (27.2%)	0 (0%)	3 (13.6%)

A total of 2192 breast reconstructions were noted to be done in 1786 patients across various centers in the UK and Europe. This includes reconstructions done from as early as November 2012 (from outcomes reported by Berna et al. [6]) to June 2020 (from outcomes reported by Mura et al. [13]).

However, it cannot be ruled out that Chandarana et al. [11] reported outcomes of pooled data across 18 centers in the UK. This could have actually included the outcomes of the case series reported by Vidya et al. [15] and Jafferbhoy et al. [5], who were also part of the nationwide Braxon® audit group.

The most common complication overall was seroma 74 (10%) (6.7%-36.3%), followed by implant loss seen in 48 (6.47%) 1.7%-27.2% cases. Major complications, including re-admission and return to theater, were seen in 98 (13.2%) 2%-27.2% reconstructions, whereas minor complications were seen in 111 (14.96%) 8%-22.7% reconstructions, including hematoma 29 (3.90%) 0-10.8%, Red breast syndrome 38 (5.12%) 0-24.4%, infection 26 (3.5%) 0%-27.2%, necrosis 26 (3.5%) 0-4.5% and wound dehiscence 24 (3.23%) 0%-11.7%.

Pre-pectoral implant-based breast reconstruction with placement of breast implant in the subcutaneous plane was developed more than four decades ago. However, interestingly, the traditional practice of implant-based breast reconstruction happens to be the sub-pectoral technique of implant placement, which evolved as a method to overcome complications seen with the former [8]. This was because the subcutaneous implant placement technique was associated with complications like capsular contracture, implant mal-positioning, and implant loss secondary to infections and other wound complications.

With the development of sub-pectoral implant-based breast reconstruction, these complications were seen to be drastically reduced, with capsular contracture rates nearly halved. The advantage of this technique over the pre-pectoral implant placement was that the implant was covered all around by the muscle. This soft tissue coverage around the implant was lacking in the pre-pectoral technique, which made it more prone to infection and other complications. Also, there were concerns about ischemia of skin flaps, loss of NAC (in case of nipple-sparing mastectomy), and eventually implant exposure in the absence of interposed muscle with subcutaneous implant placement [7].

However, in recent years, the technique of pre-pectoral implant-based breast reconstruction has been revived and re-introduced in the surgical practice. This has been possible because of the development and introduction of the use of acellular dermal matrices in breast reconstruction. Berna et al. described this muscle-sparing technique of pre-pectoral implant-based reconstruction using ADM, which provides complete coverage over the implant similar to that provided with the submuscular pocket while avoiding all the problems resulting from muscle detachment [6]. Hence, this allows the fully covered implant to be placed in the subcutaneous plane without the need to mobilize the pectoralis muscle.

In this systematic review, we analyzed eight studies, all of them being case series, reporting the short-term outcomes of pre-pectoral implant-based breast reconstruction using Braxon®. Numerous studies have included systematic reviews of the outcomes of pre-pectoral/subcutaneous implant-based breast reconstruction using acellular dermal matrices. However, these ADMs differ from one another depending on their tissue of origin or source, manufacturing and preservation techniques and their quality. Hence, there is a shortcoming in generalizing their results. Although the surgical technique is the same in all these studies, but the outcomes with each different ADM may potentially vary, owing to their individual differences.

The use of Braxon® mesh is gaining popularity all over the UK and Europe, as it is the only ADM that is approved for this specific application and comes in a pre-shaped form. Therefore, it is pertinent to study its outcomes separately for a more informed decision on its adoption in routine clinical practice [10].

While the main advantages of a muscle-sparing pre-pectoral implant-based breast reconstruction are unique to the technique itself, which prevents the post-operative pain and discomfort associated with detaching the pectoralis muscle, the acceptability of this operation also depends upon the complication and failure rates associated with the use of ADM. These, in turn, are dictated by the nature and quality of the ADM material used for covering the implant. There are various advantages perceived by Braxon® mesh, both from surgeons’ and patients’ perspectives. Firstly, the pre-shaped form of Braxon® allows for a shorter learning curve and more uniformity in technique among the surgeons [13]. Secondly, it is preservative-free and is claimed to have minimal immunogenic potential. The host immune response is believed to be the underlying cause of various complications such as seroma and Red breast syndrome.

Using the ADM to cover the implant adds an extra layer of support between the implant and the mastectomy flaps. This gets vascularized and incorporated into the subcutaneous tissue of the overlying skin flaps. The other benefit of using ADM seems to be that it spares the mastectomy skin flaps from tension by taking the weight of the enclosed implant. Because the ADM is anchored to the chest muscles, the closure of the mastectomy flaps remains relatively tension-free, allowing better wound healing. This is reflected by the low overall rates of wound dehiscence 24 (3.23%) and ischemic complications such as skin and NAC necrosis 26 (3.50%) seen in this systematic review.

As there are no studies that have directly compared outcomes of pre-pectoral implant-based reconstruction with sub-pectoral implant-based reconstruction, the outcomes could only be compared with those reported in the existing literature. One reason for this was that the use of Braxon® has replaced the sub-pectoral technique. In other words, the surgeons who are using Braxon® are no longer practicing sub-pectoral reconstructions. Hence, it is difficult to find a center where both techniques are being used, giving an opportunity to draw comparisons between the two.

The results of this systematic review of the included studies reflect that complication rates of breast reconstruction with Braxon® are low and comparable with the published evidence. These data can be compared with the results of the two large national audits from the UK, which have reported outcomes of implant-based breast reconstructions.

The complication rates in this systematic review are much less than those in these two national audits. In fact, the implant loss rate in this systematic review is 48 (6.5%), which is less than that of both these audits. Table 4 summarizes the comparison between the complication rates of the current review and these audits. It is clear that the pooled complication rates of this review reflect better outcomes than those reflected in both these audits, where the implant-based reconstructions were primarily sub-pectoral.

**Table 4:** Comparison of results with NMBRA and iBRA audits. NMBRA: National Mastectomy and Breast Reconstruction Audit, iBRA: implant-based breast reconstruction study.

Complications	NMBRA	iBRA
Re-admission rates	16	18
Return to theater	4.6	18
Implant loss	9	9
Infection	25	25

Therefore, the overall collective evidence in the published literature has shown reasonably favorable outcomes of pre-pectoral implant-based reconstruction using Braxon® ADM. This is not only in terms of safety and lesser complications but in other aspects that this review has not effectively explored, such as the overall cosmesis and patient satisfaction. Nonetheless, the long-term outcomes and the impact of adjuvant radiotherapy are yet to be analyzed.

Although pre-pectoral implant-based breast reconstruction using Braxon® mesh appears to be a promising technique with acceptable outcomes, there remains a need for further investigation. Conducting randomized controlled trials comparing Braxon® with other acellular dermal matrices (ADMs) could provide more objective conclusions and a higher level of evidence, contributing to the refinement and validation of this approach in clinical practice.

This systematic review had several limitations. No randomized control trials have been done on Braxon® Mesh so far. The included studies are retrospective analyses and will have the disadvantages of retrospective studies. Therefore, the level of evidence is not very strong, as gathered from the limited number of case series. Heterogeneity between the surgical practice in different centers and the lack of a control group (patients undergoing sub-pectoral implant-based reconstruction) for comparison could have contributed to significant confounding effects. The results of this review only reflect the short-term outcomes of the Braxon® mesh because of the short follow-up. Longer-term outcomes are yet unknown.

## Conclusions

The experience with this novel technique of pre-pectoral implant-based breast reconstruction using Braxon® mesh is still early, yet promising, as reflected in the outcomes of this review, which are comparable with those of the sub-pectoral technique. It represents a relatively painless muscle-sparing technique of breast reconstruction, providing esthetically acceptable results. Shorter operating times, faster post-operative recovery, and shortened hospital stays are the major advantages of the pre-pectoral implant placement technique. However, there is still the need for longer-term studies to report the long-term outcomes, such as capsular contracture, rippling, post-operative animation deformity, and the impact of adjuvant radiotherapy on the final cosmetic outcome.
